# Understanding the role of self in auditory verbal hallucinations using a self‐discrepancy paradigm

**DOI:** 10.1111/papt.12276

**Published:** 2020-04-13

**Authors:** Monique Scott, Susan L. Rossell, Wei Lin Toh, Neil Thomas

**Affiliations:** ^1^ Centre for Mental Health Swinburne University of Technology Melbourne Victoria Australia; ^2^ Psychiatry St Vincent's Hospital Melbourne Victoria Australia; ^3^ The Alfred Hospital Melbourne Victoria Australia

**Keywords:** hallucinations, hearing voices, psychosis, schizophrenia, self‐concept, self‐regulation

## Abstract

**Objective:**

Negative auditory verbal hallucination (AVH) content is a major predictor of distress and typically occurs more frequently than positive or neutral content. Recent research has found that negative views of self are associated with the negative content of AVHs. However, research investigating the role of self in AVH content is in its infancy and warrants further study.

**Design:**

The current study examined correspondence between negative voice content and internalized representations of self, using a self‐discrepancy paradigm incorporating multiple domains of self (ideal, ought, and feared). It also considered the impact of depression and anxiety.

**Method:**

An adapted self‐discrepancy questionnaire was administered to a transdiagnostic clinical sample of 86 current voice‐hearers. Participants rated how similar they believed themselves to be (actual self), and how similar their voices would say they are (voice self), to their ideal, ought, and feared self‐concepts.

**Results:**

Voice content was related to how the person viewed themselves in relation to their ideal, ought, and feared self‐concepts. Additionally, voices reflected negative feared self‐concepts, particularly in people with anxiety.

**Conclusions:**

These findings provide further insight into the phenomenon of hearing voices and have the potential to change the way we approach formulation and treatment of AVHs. In particular, treatment approaches that reduce discrepancies between how one views themselves and their positive and negative self‐concepts, or alter the relationship one has with their self‐concepts and negative voices, have the potential to reduce the impact of distressing voices.

**Practitioner points:**

- Voice experiences can be meaningfully related to how the person views themselves in relation to concepts of their ideal, ought, and feared selves
- Negative voice content might be understood as reflecting discrepancies from these self‐representations, which may have a self‐regulatory function in relation to goal‐directed behaviour.
- Identifying how voice content relates to self could be useful in not only challenging the extent of perceived self‐discrepancies, but also considering how to enact valued parts of self.

## Background

Auditory verbal hallucinations (AVHs), or voices, refer to perceiving verbal speech in the absence of a corresponding external stimulus (Slade & Bentall, 1988). Voices most commonly occur in schizophrenia‐related disorders, but are also known to occur in other clinical diagnoses, such as major depressive disorder and bipolar disorder (Toh, Thomas, & Rossell, 2015), as well as in a small but significant number of people with no clinical diagnosis (Sommer *et al.*, 2010). The majority of voice content tends to be negative, and negative content is of clinical importance as it is associated with greater distress than content that is neutral or positive (Woodward *et al.*, 2014). Common negative voice content includes critical, threatening, commanding, and persecuting voices. Voices that are critical in content are especially common (Nyani & David, 1996). Given this, it is plausible that models of self‐criticism may be of value when trying to understand underlying mechanisms in negative AVH content. This idea is in accordance with widely accepted cognitive models of voice‐hearing, which propose that people who hear voices tend to misattribute internally generated signals, such as inner speech, as being external (Allen, Aleman, & McGuire, 2007; Bentall, 1990).

Many models of self‐criticism suggest that self‐critical thoughts stem from negative concepts of the self (Beck, 2011; Young, Klosko, & Weishaar, 2003). This is relevant to hearing voices, as the negative beliefs people hold about oneself have been proposed to manifest in their voice content (Beck & Rector, 2005; Close & Garety, 1998; Paulik, 2012). Indeed, extreme negative evaluations of the self are common in people with psychosis (Fowler *et al.*, 2006; Gracie *et al.*, 2007; Noone *et al.*, 2015; Smith *et al.*, 2006; Thomas, Farhall, & Shawyer, 2015), and negative beliefs about the self are associated with hearing voices in clinical (Barrowclough *et al.*, 2003; Close & Garety, 1998) and non‐clinical populations (Bortolon, Seille, & Raffard, 2017). Moreover, more negative views of self correspond to hearing voices rated as more negative, frequent, and distressing (Smith *et al.*, 2006). In addition, the relationship between negative self‐beliefs and voices has been shown to be mediated by negative affect (Jaya, Ascone, & Lincoln, 2017), which suggests that emotion may contribute to the relationship between negative self‐concept and hearing negative voices. However, research investigating the role of self in AVH content is still in its infancy, and further study is warranted. Prominent models of self, such as the self‐discrepancy model, offer a framework for gaining further insights into the relationship between self and AVH content.

The self‐discrepancy model proposes that multiple domains of self can be operationalized within the self‐concept, including how a person perceives oneself to be (*actual self*) and concepts of how a person could possibly be: These include how they would ideally like to be (*ideal self*), ought to be (*ought self*; Higgins, Bond, Klien, & Strauman, 1986), and fear being (*feared self*; Carver, Lawrence, & Scheier, 1999). Self‐discrepancies have typically been measured using questionnaires asking people to list a number of attributes for each of their self‐domains, and calculating synonyms and autonyms to obtain a total discrepancy score (Higgins, Klein, & Strauman, 1985), or having respondents rate the discrepancy between their actual self‐domain and other possible self‐domains using Likert scales (Hardin & Lakin, 2009; Strauman & Higgins, 1987). This may be from an individual's own personal standpoint or that of a significant other, for example, mother, father, sibling, partner, and close friend (Higgins *et al*., 1986). As such, discrepancy refers to the distance between the actual self‐ and another possible domain of self (ideal, ought, or feared).

The key developmental premise of self‐discrepancy theory is that representations of self are acquired during childhood through positive and negative emotional and social experiences, thereby establishing internalized standards for self‐evaluation (Higgins, 1999). These internal representations of self may have evolved to guide self‐regulation and motivate goal‐directed behaviour, where self‐regulation operates through a process of self‐monitoring, judgement of self in relation to personal standards or goals, and one's affective response (social cognitive theories of self‐regulation; Bandura, 1991). Thus, self‐standards may directly prompt action and, through their use in self‐evaluation, arouse emotions that are themselves motivating. Similarly, perceptual control theory theorizes that self‐regulation occurs through a discrepancy‐reducing negative feedback process that functions to minimize disparities between one's perceived value, possibly their self‐concept, and other internal standards or goals, such as self‐aspirations (Powers, 2005). To this end, critical voice content may potentially serve a self‐regulatory function, arising in order to resolve discrepancies between actual and preferred (ideal/ought) self‐states or reduce consistency with feared self‐states. This idea is in line with propositions that voice content arises from developmentally immature inner speech processes primarily directing behaviours towards action (Fernyhough, 2004; Vygotsky, 1980), and finds support from preliminary evidence that voice content may serve a functional purpose towards goal‐directed behaviour (Varese, Tai, Pearson, & Mansell, 2016).

As such, studies have demonstrated that discrepancies between one's actual and other domains of self can be associated with psychopathology (Carver *et al.*, 1999; Higgins, 1983, 1987, 1999; Higgins *et al.*, 1985). Specifically, discrepancies between actual and ideal selves have been found in depression, and discrepancies between actual and ought selves have been found in anxiety (Hardin & Lakin, 2009; Higgins *et al*., 1986; Higgins, 1987, 1999; Scott & Ohara, 1993; Strauman, 1989; Strauman & Higgins, 1987). Additionally, consistencies (i.e., low discrepancy) between actual and feared selves have been found to be related to both depression and anxiety (Carver *et al.*, 1999). Similarly, people experiencing depressive episodes in bipolar disorder have been shown to demonstrate higher consistency with their feared self than non‐clinical controls (Alatiq, Crane, Williams, & Goodwin, 2010), whereas consistency between actual and ideal or ought selves has been found in persons with mania (Alatiq *et al.*, 2010; Bentall, Kinderman, & Manson, 2005). One explanation for these findings can be found in mood congruency theory, whereby people tend to hold and recall beliefs congruent to their mood (Bower, 1981; Chatterjee & Kulhara, 1989; Delgado & Chaves, 2013; Upthegrove *et al.*, 2015; Winokur, Scharfetter, & Angst, 1985). As such, people may be more likely to rate oneself as being more similar to their positive self‐concepts (ideal and ought selves) and discrepant from their negative self‐concepts (feared self) when experiencing positive emotions, and conversely, be more likely to rate oneself as being more similar to their negative selves and discrepant from their positive selves when experiencing negative emotions.

The aim of the current study was to investigate the relationship between self‐discrepancies, perceived views of self from the perspective of voices, negative voice content, and emotional state (depression and anxiety). The use of a self‐discrepancy paradigm presents a unique and novel approach for investigating the role of self in hearing voices, allowing us to explore how multiple domains of self may manifest in voice content. To do this, the current study investigated three discrepancies from the standpoint of self: actual–ideal, actual–ought, and actual–feared, as well as three discrepancies from the standpoint of one's voices: voice–ideal, voice–ought, and voice–feared. Self‐discrepancy was operationalized as the magnitude of difference between actual and other possible domains of self (ideal, ought, and feared) based on one's own standpoint. Voice discrepancy was operationalized as the magnitude of difference between voice content about the self and other possible domains of self (ideal, ought, and feared) based on the standpoint of one's voices. For ease of reference, definitions of these key terms are presented in Table 1.

**Table 1:** Definition of important terms in our self‐discrepancy model

Term	Definition
Actual self	How a person believes oneself to actually be
Ideal self	Traits of the kind of person one would ideally like to be
Ought self	Traits of the kind of person one believes one ought to be
Feared self	Traits of the kind of person one would fear being
Voice self	Traits of the kind of person one's voices would say they are
Actual–ideal discrepancy	Magnitude of discrepancy between how one believes oneself to actually be and one's ideal self‐traits
Actual–ought discrepancy	Magnitude of discrepancy between how one believes oneself to actually be and one's ought self‐traits
Actual–feared discrepancy	Magnitude of discrepancy between how one believes oneself to actually be and one's feared self‐traits
Voice–ideal discrepancy	Magnitude of discrepancy between how one's voices would say they are and ideal self‐traits
Voice–ought discrepancy	Magnitude of discrepancy between how one's voices would say they are and their ought self‐traits
Voice–feared discrepancy	Magnitude of discrepancy between how one's voices would say they are and their feared self‐traits
Self‐discrepancies	Discrepancies between how a person believes oneself to actually be (actual self) from one's ideal, ought, and feared selves
Voice discrepancies	Discrepancies between how one's voices would say one is (voice self) from one's ideal, ought, and feared selves

The following hypotheses were proposed.

Hypothesis 1: Based on observations that voice content is typically negative and critical in psychotic disorders (Larøi *et al.*, 2019), it was hypothesized that voice–feared discrepancies would be smaller than voice–ideal and voice–actual discrepancies. In other words, the representation of self as the voice says the person is would be closer to their feared than their ideal or ought self‐domains.

Hypothesis 2: Based on findings that negative beliefs about self are related to negative AVH content (Barrowclough *et al.*, 2003; Close & Garety, 1998), it was hypothesized that actual–ideal, actual–ought, and actual–feared discrepancies would predict voice–ideal, voice–ought, and voice–feared discrepancies, respectively.

Hypothesis 3: In accordance with previous findings of associations between self‐discrepancies and negative emotional states (depression, anxiety), it was expected that depression and anxiety would predict lower voice–feared discrepancies. In other words, the representation of self as the voice says the person is would be closer to their feared self in people with higher depression and anxiety scores.

Hypothesis 4: Based on findings that people with affective psychosis experience more negative voice content than those with non‐affective psychosis (Chatterjee & Kulhara, 1989; Delgado & Chaves, 2013; Upthegrove *et al.*, 2015; Winokur *et al.*, 1985), it was predicted that voice–feared discrepancy would be lower in affective psychosis than in non‐affective psychosis. In other words, the representation of self as the voice says the person is would be closer to their feared self in people with affective psychosis than in people with non‐affective psychosis.

## Methods

### Participants and procedure

Participants were recruited as part of a broader study on voice phenomenology (Rossell *et al*., 2019). They included 86 people with current voices (occurring within the last 14 days) and a psychiatric diagnosis of schizophrenia, schizoaffective disorder, major depressive disorder, or bipolar disorder. Participant's diagnosis was confirmed using the Mini International Neuropsychiatric Interview Screen 5.0. (MINI, Sheehan *et al.*, 1998). Exclusion criteria included people with current substance use disorder, substance‐induced psychosis, Wechsler Test of Adult Reading (WTAR; Holdnack, 2001) Full Scale IQ below 70, and acquired brain injury; individuals with significant neurological disorders (e.g., Parkinson's disease); and persons who had undergone electroconvulsive therapy within the last 6 months.

Participants were recruited from the inpatient and outpatient services of two public and one private psychiatric hospital, from a participant registry associated with a specialist Voices Clinic, and from online and print advertising, in Melbourne, Australia. Ethics approvals were received from requisite institutions. Participants provided written informed consent, and procedures were in line with the Declaration of Helsinki (World Medical Association, 2013). Clinical interviews were conducted by one of three trained researchers.

### Measures

*Demographic and clinical information* collected included gender, age, primary language, country of birth, education, employment, medical history, and alcohol and substance consumption.

#### Psychotic Symptom Rating Scales

The Psychotic Symptom Rating Scales (PSYRATS; Haddock, McCarron, Tarrier, & Faragher, 1999) was used to quantify dimensions of voice experience. The factor structure described by Woodward *et al*. (2014) was used to calculate factor scores.

#### Beck Anxiety Inventory

The Beck Anxiety Inventory (BAI; Beck, Epstein, Brown, & Steer, 1988) is a 21‐item self‐report measure of anxiety severity. Items are scored on a 0–3 scale and summed to generate a total score ranging from 0 to 63, with higher scores indicating more severe anxiety symptomatology.

#### Beck Depression Inventory‐II

The Beck Depression Inventory‐II (BDI‐II; Beck, Steer, & Brown, 1996) is a 21‐item self‐report measure of depressive symptomatology. Items are scored on four‐point scales and summed to create a total score ranging from 0 to 63, with higher scores indicating more severe depressive symptoms.

#### Young Mania Rating Scale

The Young Mania Rating Scale (YMRS; Young, Biggs, Ziegler, & Meyer, 1978) is an 11‐item rating scale for manic symptoms based on a clinical interview producing a score of 0–60.

#### Selves Questionnaire for Voices

The Selves Questionnaire for Voices (SQV) was an experimental measure adapted from the Selves Questionnaire (Carver *et al.*, 1999; Higgins *et al*., 1985) for use in this study. It was adapted to include a measure of a voice self to use for comparison with the other possible self‐domains (ideal, ought, and feared selves). Respondents were asked to list five traits each of their ideal, ought, and feared self‐domains. They could either generate their own terms or use the prompt sheet provided containing 181 trait terms (obtained from the Integrated Self‐Discrepancy Index; Hardin & Lakin, 2009). Participants were then asked to rate how similar they believed they actually were (actual self) to each of their self‐listed traits, and how similar their voice(s) would say they were (voice self) to each of the traits. Ratings were made on Likert scales ranging from 1 (opposite of this trait) to 7 (just like this trait). Thus, participants generated their own reports of similarities/discrepancies with respect to ideal, ought, and feared selves. Scores between the actual self‐ and other possible self‐domains (ideal, ought, and feared) and between the voice self‐ and other possible self‐domains (ideal, ought, and feared) were tallied to obtain total similarity scores. These scores were then reverse‐scored, and means were calculated to obtain discrepancy scores between actual–ideal, actual–ought, actual–feared, voice–ideal, voice–ought, and voice–feared selves, making up the six subscales on the SQV. Higher scores indicated higher discrepancy, and lower scores indicated lower discrepancy (i.e., consistency). In addition, respondents were asked to list five of their ‘voice self’ traits, operationalized as ‘the kind of person their voice(s) would say they are’. This was used for descriptive purposes only, and not for calculating discrepancy scores. Psychometric properties of the SQV can be found in Table 2.

**Table 2:** Participant characteristics and descriptive statistics for main measures and subscales

	Mean (*SD*)	Range	
Age	38.06 (13.21)	19–65	
Age of illness onset	23.35 (9.76)	5–50	
Age of auditory verbal hallucination onset	21.24 (10.96)	3–49	
Estimated premorbid IQ	104.42 (11.87)	71–124	

	*n* (%)		
Gender			
Female	51 (59%)		
Male	35 (41%)		
Diagnostic group			
Schizophrenia	36 (42%)		
Schizoaffective disorder	14 (16%)		
Bipolar disorder	19 (22%)		
Major depressive disorder	17 (20%)		
Inpatients versus outpatients			
Inpatients	15 (17%)		
Outpatients	71 (83%)		
Antipsychotic use	46 (54%)		
English as first language	77 (90%)		
Born in English‐speaking country	74 (86%)		

	Mean (*SD*)	Range	α
SQV actual–ideal discrepancy	14.17 (5.94)	4.2–29.4	.78
SQV actual–ought discrepancy	14.60 (5.99)	4.2–29.4	.80
SQV actual–feared discrepancy	20.13 (6.26)	4.2–29.4	.84
SQV voice–ideal discrepancy	20.46 (7.64)	4.2–29.4	.90
SQV voice–ought discrepancy	19.78 (7.87)	4.2–29.4	.92
SQV voice–feared discrepancy	13.58 (7.22)	4.2–29.4	.90
BDI‐II depression	21.79 (13.57)	0–54	.93
BAI anxiety	23.58 *(*13.75*)*	0–63	.92
PSYRATS distress	11.55 (4.83)	3–20	.66
PSYRATS frequency	5.04 (2.72)	0–12	.76
PSYRATS attribution	4.63 (1.97)	0–8	.41
PSYRATS loudness	1.80 (0.79)	0–4	

Notes

α = Cronbach's alpha; BAI = Beck Anxiety Inventory; BDI‐II = Beck Depression Inventory‐II; PSYRATS = Psychotic Symptom Rating Scales; SQV = Selves Questionnaire for Voices.

### Statistical analyses

Statistical analyses were conducted using SPSS v.26 (IBM Corp., Armonk, NY, USA). Descriptive statistics were obtained, including calculating the 10 most frequently listed trait terms regarding ideal, ought, feared, and voice selves. To address hypothesis 1, paired‐sample *t*‐tests were performed to assess differences in voice discrepancies. To address hypothesis 2, correlation analyses were performed to investigate the relationships between self‐discrepancies and voice discrepancies, using Bonferroni correction to control for Type I errors. To test hypothesis 3, a linear regression analysis was performed to estimate the proportion of variance in voice–feared discrepancy that may be accounted for by depression and anxiety. For hypothesis 4, an independent‐samples *t*‐test was performed to examine the differences between voice–feared discrepancy in affective versus non‐affective psychosis.

## Results

### Participant characteristics and descriptive statistics

Table 2 presents basic demographic and clinical characteristics, along with mean scores on the mood and self‐discrepancy outcome measures. The majority of participants (*n* = 72, 84%) reported at least some negative voice content in the last week, with 49 (57%) reporting that the majority of content was negative. Nine participants had manic symptoms (YMRS ≥ 12). A series of *t*‐tests revealed no statistical differences in voice–ideal, voice–ought, or voice–feared discrepancy scores on the basis of manic versus non‐manic presentation (*p *> .05), or on PSYRATS distress, PSYRATS degree of negative content, or BDI and BAI scores (*p *> .05), so these participants were included in remaining analyses.

Ratings of self‐ and voice discrepancies, relative to representations of the ideal, ought, and feared selves, showed good internal consistencies. Voice–ideal (*r *= .33, *p *< .001), voice–ought (*r *= .26, *p *= .01), and voice–feared (*r *= −.30, *p *< .001) discrepancies were each correlated with the voice distress factor of the PSYRATS. No other correlations were observed between the SDQ and PSYRATS scales.

### Traits of ideal, ought, feared, and voice selves

A total of 517 different trait terms were listed by participants, 146 of which were words listed on the prompt sheet provided. There was a total of 176 different traits listed for the ideal self, 183 for the ought self, 185 for the feared self, and 243 for the voice self. Table 3 presents the most frequently listed trait terms by participants regarding their ideal, ought, feared, and voice selves. Note that synonyms and related terms were not grouped together, and this list simply reflects the most frequently listed trait terms listed by participants for each self‐domain.

**Table 3:** Ten most common trait terms generated by participants for ideal, ought, feared, and voice selves

Ideal self	*n*	Ought self	*n*	Feared self	*n*	Voice self	*n*
Kind	22	Kind	18	Aggressive	18	Stupid	13
Happy	17	Intelligent	12	Lazy	16	Lazy	9
Creative	14	Trustworthy	11	Selfish	14	Ugly	8
Intelligent	12	Helpful	11	Mean	13	Kind	7
Friendly	10	Friendly	9	Bossy	10	Troublesome	6
Artistic	9	Caring	8	Angry	9	Aggressive	6
Helpful	7	Punctual	8	Unreliable	8	Selfish	6
Successful	7	Optimistic	8	Childish	8	Mean	4
Reliable	6	Mature	8	Untruthful	7	Worthless	4
Relaxed	6	Logical	6	Pessimistic	7	Pessimistic	4
Mature	6					Overconfident	4
Generous	6					Gullible	4
Confident	6					Neurotic	4
Cheerful	6						

Note

*n* = number of times the trait was listed by participants. Where multiple trait terms were the tenth most frequently listed, all trait terms with that frequency were listed.

### Differences in the discrepancies between voice and feared selves, voice and ideal selves, and voice and ought selves

Results of paired‐sample *t*‐tests showed that as predicted, voice–feared discrepancy was significantly lower than voice–ideal discrepancy, *t*(83) = −5.04, *p *< .01, and voice–ought discrepancy, *t*(80) = −4.09, *p *< .01.

### Relationship between self‐discrepancies (actual–ideal, actual–ought, and actual–feared) and voice discrepancies (voice–ideal, voice–ought, and voice–feared)

Table 4 presents the correlations among the six subscales of the SQV. Voice discrepancies followed an expected pattern of intercorrelation, with voice–ideal and voice–ought discrepancies being positively correlated with each other, and each being negatively correlated with voice–feared discrepancy. The medium‐sized intercorrelations supported these being considered as independent constructs.

**Table 4:** Correlations between self‐discrepancies (actual–ideal, actual–ought, and actual–feared) and voice discrepancies (voice–ideal, voice–ought, and voice–feared)

	Voice–ideal discrepancy	Voice–ought discrepancy	Voice–feared discrepancy
Actual–ideal self‐discrepancy	.38**	.27**	−.21
Actual–ought self‐discrepancy	.24	.35**	−.02
Actual–feared self‐discrepancy	.12	−.05	.32**

Notes

*N* = 86.

** *p* < .001.

It was noted that actual–ideal and actual–ought self‐discrepancies were correlated with each other, but both were independent of actual–feared self‐discrepancy.

In considering the hypothesized relationship between actual and voice self‐ratings, relative to ideal, ought, and feared domains, the expected pattern of relationships was observed: Actual–ideal discrepancy was significantly related to voice–ideal discrepancy, actual–ought discrepancy was significantly related to voice–ought discrepancy, and actual–feared discrepancy was significantly related to voice–feared discrepancy.

### The relationship between voice–feared discrepancy and negative mood state

To determine whether variance in voice–feared discrepancy could be accounted for by depression and anxiety, a linear regression analysis was performed. Results showed that in combination, higher anxiety and depression ratings predicted lower voice–feared discrepancies, *R* ^2^ = .09, adjusted *R* ^2^ = .07, *F*(2, 78) = 3.89, *p* = .02. However, anxiety was the only significant individual predictor of voice–feared discrepancy, and depression did not independently contribute to the regression model. Unstandardized and standardized regression coefficients and squared semi‐partial correlations for each predictor in the regression model are reported in Table 5.

**Table 5:** Unstandardized (*B*) and standardized regression coefficients (β), and squared semi‐partial correlations (sr2) for each predictor in a regression model predicting voice–feared discrepancy

Variable	*B* (95% CI)	β	sr^2^	*t*	*p*
Depression	−0.03 (−0.16, 0.11)	−.05	−.04	−0.37	.72
Anxiety	−0.14 (−0.28, −0.01)	−.27	−.23	−2.10	.04

Notes

*N* = 86.

CI = confidence interval.

Results of the *t*‐test to examine whether there were differences by diagnosis showed that voice–feared discrepancy did not differ between affective (*M* = 13.21, *SD* = 7.34) versus non‐affective (*M* = 13.84, *SD* = 7.20) groups, *t*(83) = −0.40, *p *= .69.

## Discussion

The purpose of this study was to investigate the relationship between domains of self and negative voice content using a novel self‐discrepancy paradigm. Participants were able to successfully elicit trait terms for their ideal, ought, and feared selves, as in the standard self‐discrepancy task, and were also able to rate how their voices would say they were in terms of these generated traits, producing a measure of self according to one's voices. The results supported the first hypothesis that across the sample, ratings of how one's voices would say they are would be discrepant from positive (ideal and ought) self‐concepts and consistent with negative (feared) self‐concepts. This speaks to the negative and critical nature of voice content as evidenced in AVH phenomenology studies (McCarthy‐Jones *et al.*, 2014; Nyani & David, 1996).

The results also supported the second hypothesis that discrepancies between how a person's voices would say they are to their ideal, ought, and feared selves mirrored actual–ideal, actual–ought, and actual–feared self‐discrepancies. This suggests that experiences of voices can be meaningfully related to multiple domains of self (ideal, ought, and feared) and that people with greater positive and lower negative self‐discrepancies may experience more negative voices. These findings build upon previous research using measures of beliefs about self in providing evidence of a relationship between negative views of self and negative voice content, whereby more negative views of self are related to greater negative voice content, in turn associated with greater voice‐related distress (Close & Garety, 1998; Paulik, 2012; Smith *et al.*, 2006).

It should be noted that these data are cross‐sectional, and it is not clear whether these elements of self‐concept are causal in generating more negative voices. Potentially, a negative view of the self could be a consequence of hearing consistently critical voices. Longitudinal research would be required to explore this relationship further.

If internal representations of self have evolved to motivate goal‐directed behaviour (Varese *et al.*, 2016), utilizing a self‐discrepancy paradigm may provide a means for measuring such goals, as in the ideal and ought self. Moreover, if self‐regulation occurs through a discrepancy‐reducing negative feedback process that functions to minimize differences between one's sensed value and some other standard (Powers, 2005; Weiner, 1948), the existence of a feared self is likely serving a self‐regulatory function. Thus, if one's voices are telling them that they are similar to a concept of how they would not want to be (feared self) and dissimilar to how they would want to be (ideal and ought self), voice content may be a manifestation of psychological processes that have evolved to facilitate self‐regulation, in a similar way that inner speech in self‐criticism and rumination may have (Powers, Koestner, & Zuroff, 2007). Though, in voice‐hearing experiences, these developmentally immature inner speech processes (Fernyhough, 2004; Vygotsky, 1980) may have been misattributed (Allen *et al.*, 2007; Bentall, 1990; Jones, 2010).

In addition, taking a view of voices as audible attempts to self‐regulate aligns with voice‐hearer accounts of AVHs as helpful, despite comprising predominantly negative content. For example, in qualitative research by Romme and Escher (2010), one voice‐hearer explained: ‘when the voices said to me “look at her, what a disaster”, I looked in the mirror and thought “they are right, I should dress properly”, and thus from a negative influence it became a stimulus’.

Furthermore, having participants generate a conceptualization of a voice self (i.e., how one's voices would say they are) provided insightful information into what voices actually say about the self. There appeared to be thematic associations between traits of the feared self‐domain and AVH content, as generated in the conceptualization of a voice self (i.e., how one's voices would say they are). Ugly and lazy were the two most commonly generated traits. This is relevant to a recent study that found that voices commenting about appearance is extremely typical, and possibly due to the high prevalence of weight gain resulting from inactivity and medication side effects in psychosis (Marshall, Freeman, & Waite, 2019). The third most commonly generated trait within the voice self was stupid, notably the opposite of intelligent, which was a frequently generated trait in ideal and ought self‐concepts. Positive traits in the voice self were also generated, such as kind, suggesting that voices may also be related to how one would ideally like to be or believe they ought to be. Along with being consistent with voice phenomenology research that voice‐hearers commonly experience a combination of negative, positive, and neutral voices (Nyani & David, 1996), these findings reinforce the notion of voices acting as agents for motivating goal‐directed behaviour and serving a self‐regulatory function.

This study also made a number of predictions pertaining to the interactions between mood state and voice consistency with feared self‐concepts. The results partially supported the third hypothesis that symptoms of depression and anxiety would predict higher consistencies between voice and feared self‐concepts; anxiety was the only significant independent predictor. These findings suggest that anxiety is associated with voices saying the person is similar to the version of oneself they most fear to be. This mirrors previous findings by Carver *et al*. (1999), where consistencies between actual and feared self‐concepts were found to be significantly related to anxiety in non‐clinical persons. Moreover, voice consistency with the feared self‐concept, particularly in anxiety, suggests that voices may be reflecting a great proportion of critical content relating to one's self‐concept, underpinned by anxious mood states. This is consistent with research demonstrating a relationship between self‐criticism and anxiety (Werner, Tibubos, Rohrmann, & Reiss, 2019) and with voice phenomenology research demonstrating that a large proportion of voice content is critical (McCarthy‐Jones *et al.*, 2014; Nyani & David, 1996).

The fourth hypothesis that the magnitude of consistency between voice and feared self‐concepts would be higher in persons with affective psychosis (bipolar disorder or major depressive disorder) than in people with non‐affective psychosis (schizophrenia or schizoaffective disorder) was not supported. This indicates that voices seem equally likely to say individuals are similar to their negative self‐concepts in non‐affective psychosis as in affective psychosis. While other studies have found negative voice content is more common in affective than in non‐affective psychosis (Chatterjee & Kulhara, 1989; Delgado & Chaves, 2013; Upthegrove *et al.*, 2015; Winokur *et al.*, 1985), some studies have found similar (Okulate & Jones, 2003) or greater rates of negative content (Kumari, Chaudhury, & Kumar, 2013). Our findings suggest that when voice content is more specifically considered in terms of alignment with negative self‐concept, consistency is apparent transdiagnostically. This would be consistent with other studies that have observed associations between symptoms and both schemas and affect in schizophrenia‐related disorders (Jaya *et al.*, 2017; Smith *et al.*, 2006).

### Clinical implications

In terms of clinical implications, establishing these relationships between voice experiences and broader self‐concept highlights the potential importance of working with self in psychological therapies for voices. To do this, a traditional focus has been to target negative beliefs about self via cognitive approaches that attempt to modify maladaptive self‐beliefs (e.g., Morrison & Renton, 2001) and/or strengthen positive self‐beliefs (e.g., Hall & Tarrier, 2003). Competitive memory training has also been used to reinforce memories opposing negative voice content (van der Gaag, van Oosterhout, Daalman, Sommer, & Korrelboom, 2012). The alternative approach of letting go of attachment to self‐concept has also formed part of broader mindfulness‐ (e.g., Chadwick, 2006; Louise, Rossell, & Thomas, 2018), acceptance‐ (e.g., Thomas, Morris, Shawyer, & Farhall, 2013), and compassion‐based (Heriot‐Maitland, McCarthy‐Jones, Longden, & Gilbert, 2019) approaches.

The use of a self‐discrepancy model further suggests that negative voice content might be conceptualized as manifestations of attempts to correct deviation from high‐level goals (ideal self and ought self). This understanding may aid shared formulation of the function of voices, corresponding to some voice‐hearer accounts of the content of their voices being meaningful signals of concerns in the person's life. Operationalization of these self‐constructs may aid articulation of valued elements of how the person would like to be, allowing discrepancy to be targeted not only by challenging the extent of perceived self‐discrepancies, but also by considering how to enact valued parts of self.

While this type of approach has yet to be systematically examined, it is in line with therapeutic applications of perceptual control theory (the method of levels), where bringing a person's awareness to their internal goals, and conflicts between these and their self‐concept may re‐establish emotional equilibrium and reduce unwanted perceptual experiences (Tai, 2009). It is notable that this provides a means of approaching critical voices not exclusively as negative self‐related content to be challenged or ignored, but as related to a functional aspect of self. This may be facilitative of a broader process of adapting to living with voices through acceptance and integration as opposed to a typically antagonistic internal dynamic characterized by perceived voice malevolence and attempts to resist the experience. It would be important to develop an evidence base for this type of approach, which would be in line with a broader move towards testing transdiagnostic symptom‐targeted approaches for psychotic experiences in the literature (Thomas *et al.*, 2014).

### Limitations

Limitations include the following. First, the predominance of female participants may reflect a bias in participant self‐selection. A degree of caution should be taken when generalizing the findings to male voice‐hearers. Second, the SQV is a novel and not yet validated measure. Thus, it is unclear the extent to which it relates to voice content as opposed to perceptions of voices. Third, the use of a novel experimental measure limits opportunities for direct comparison with other studies that may have employed different measures of self‐concept. Fourth, while using a self‐discrepancy paradigm allowed for the measurement of critical voices (i.e., negative voices about the self), it did not allow for the measurement of other subtypes of negative voice content, such as threatening or commanding content. Fifth, results from independent‐samples t‐tests and correlation procedures may have been underpowered due to sample size (Cohen, 1992). Thus, subtle relationships between voice and actual discrepancies, and differences between groups on the basis of emotional state and diagnosis, although not detected in this study, may be possible.

### Directions for future research

Future research should continue investigating the role of self in AVH experiences. The self‐discrepancy approach could be extended by validation studies to determine whether the SQV is a reliable and valid measure of voice content, or alternatively perceptions about voices. Furthermore, the provision of voice self‐traits by participants in this study provided rich and novel information about what voices say about voice‐hearers, which could be a focus for future study.

As the results of this study add to the growing body of evidence implicating the role of self in voices, treatment trials that aim to specifically target self‐concept as a means of reducing the impact of critical voices could be a promising area for future research. Moreover, studies investigating the impact of more focused intervention protocols targeting self‐discrepancies and voice discrepancies would inform the utility of this as an alternative stand‐alone intervention or as a potential component of formulation‐based cognitive behavioural therapy. This would also provide a further test of self‐discrepancy as a psychological process associated with negative voice content, in line with an interventionist‐casual approach (Freeman, 2011; Thomas *et al.*, 2014). Additionally, there is limited research on the possible factors that may underpin various other types of voices (i.e., commanding and threatening), and this would be an important avenue for future research in an effort to develop further insights and targeted and effective treatments for voices.

Researchers are also encouraged to further investigate the role of emotion, particularly anxiety, in voice content: Few studies have focused on the interaction between voice content and anxiety specifically. In addition, longitudinal research could provide insights into the directional nature of the relationship between self‐beliefs and voice content. Finally, possible mechanisms that may be relevant to both concepts of self and experiences of voices include childhood trauma, attachment (Berry, Fleming, Wong, & Bucci, 2017; Berry, Wearden, Barrowclough, Oakland, & Bradley, 2012), dissociation (Berry *et al.*, 2017; Larøi *et al.*, 2019), hypervigilance, reduced social rank, altered emotional processing, and inner speech. Examination of these in relation to both voices and concepts of self is a worthy area for continuing study.

## Conflicts of interest

All authors declare no conflict of interest.

## Author contributions

Monique Scott (Conceptualization; Data curation; Formal analysis; Funding acquisition; Investigation; Methodology; Project administration; Writing – original draft); Susan L. Rossell (Conceptualization; Funding acquisition; Supervision; Writing – review & editing); Wei Lin Toh (Conceptualization; Data curation; Project administration; Writing – review & editing); Neil Thomas (Conceptualization; Methodology; Supervision; Writing – review & editing).

## Data Availability

Participants did not grant consent for inclusion of their data in a public access data repository, but data can be made accessible within the bounds of participant consent upon reasonable request.
